# Nanosecond pulsed electric field inhibits proliferation and induces apoptosis in human osteosarcoma

**DOI:** 10.1186/s13018-015-0247-z

**Published:** 2015-07-07

**Authors:** Xudong Miao, Shengyong Yin, Zhou Shao, Yi Zhang, Xinhua Chen

**Affiliations:** The Department of Orthopedics, the Second Affiliated Hospital, Zhejiang University, Hangzhou, Zhejiang Province 310003 China; The Department of Hepatobiliary and Pancreatic Surgery, the First Affiliated Hospital, Zhejiang University, Collaborative Innovation Center for Diagnosis Treatment of Infectious Diseases, 79 Qinchun Road, Hangzhou, Zhejiang Province 310003 China; The Department of Gynecology, The First Affiliated Hospital of Zhejiang Chinese Medical University, Hangzhou, Zhejiang Province 310000 China

**Keywords:** Osteosarcoma, MG-63 cells, Nanosecond pulsed electric field, Apoptosis

## Abstract

**Objective:**

Recent studies suggest that nanosecond pulsed electric field (nsPEF) is a novel minimal invasive and non-thermal ablation method that can induce apoptosis in different solid tumors. But the efficacy of nsPEF on bone-related tumors or bone metastasis is kept unknown. The current study investigates antitumor effect of nsPEF on osteosarcoma MG-63 cells in vitro.

**Method:**

MG-63 cells were treated with nsPEF with different electric field strengths (0, 10, 20, 30, 40, and 50 kV/cm) and different pulse numbers (0, 6, 12, 18, 24, and 30 pulses). The inhibitory effect of nsPEF on the growth of MG-63 cells was measured by Cell Counting Kit-8 (CCK-8) assay at different time points (0, 3, 12, 24, and 48 h post nsPEF treatment). The apoptosis was analyzed by Hoechst stain, in situ terminal deoxynucleotidyl transferase (TdT)-mediated dUTP nick-end labeling (TUNEL), and flow cytometric analysis. The expression of osteoprotegerin (OPG), receptor activator of NF-κB ligand (RANKL), and tumor necrosis factor α (TNF-α) was examined by reverse-transcription polymerase chain reaction (RT-PCR) and western blot.

**Results:**

The CCK-8 assay showed that nsPEF induced a distinct electric field strength- and pulse number-dependent reduction of cell proliferation. For treatment parameter optimizing, the condition 40 kV/cm and 30 pulses at 24 h post nsPEF achieved the most significant apoptotic induction rate. Hoechst, TUNEL, and flow cytometric analysis showed that the cell apoptosis was induced and cells were arrested in the G0/G1 phase. PCR and western blot analysis demonstrated that nsPEF up-regulated OPG expression had no effect on RANKL, increased OPG/RANKL ratio.

**Conclusion:**

NsPEF inhibits osteosarcoma growth, induces apoptosis, and affects bone metabolism by up-regulating OPG, indicating nsPEF-induced apoptosis in osteosarcoma MG-63 cells. NsPEF has potential to treat osteosarcoma or bone metastasis. When nsPEF is applied on metastatic bone tumors, it might be beneficial by inducing osteoblastic differentiation without cancer proliferation. In the future, nsPEF might be one of the treatments of metastatic bone tumor.

## Introduction

Osteosarcoma is a malignant bone tumor with high occurrence in children and young adolescents. Retrospective review showed that in the past 30 years, osteosarcoma had a poor prognosis and there was no significant improvement of disease-free survival and the stagnated situation has not improved even with the aggressive use of neoadjuvant chemotherapy and radiation therapy [1]. Patients did not benefit from overtreatment, and as a result, a high rate of lung metastasis, recurrence, and pathological fracture frequently occur, keeping osteosarcoma still one of the lowest survival rates in pediatric cancers [2]. Thus, new therapeutic strategy needs to be developed.

Nanosecond pulsed electric field (nsPEF) is an innovative electric ablation method based on high-voltage power technology, which came into medical application in the last decade [3]. NsPEF accumulates the electric field energy slowly and releases it into the tumor in ultra-short nanosecond pulses, altering electrical conductivity and permeability of the cell membrane, causing both cell apoptosis and immune reaction [4].Quite different from any other traditional local ablation method, nsPEF accumulate less Joule heating and showed no hyperthermic effects [5], indicating unique advantage over other thermal therapies such as radiofrequency, cryoablation, microwave, and interstitial laser; nsPEF can be used alone and so avoid the side effect caused by chemotherapy or percutaneous ethanol injection [6].

We have used nsPEF to ablate tumor and showed the equal outcome as the radical resection with proper indication [7]. Clinical trials and pre-clinical studies from different groups proved that nsPEF has direct antitumor effects by inhibiting proliferation and causing apoptosis in human basal cell carcinoma [8, 9], cutaneous papilloma, squamous cell carcinoma [10], melanoma [11, 12], hepatocellular tumor [13], pancreatic tumor [14], colon tumor [15, 16], breast cancer [17, 18], salivary adenoid cystic carcinoma [19], oral squamous cell carcinoma [20], et al. Local ablation with nsPEF indicates the noticeable advantage of not only eliminating original tumors but also inducing an immune reaction, e.g., enhance macrophage [21] and T cell infiltration [22] and induce an immune-protective effect against recurrences of the same cancer [23]. The characteristic of electric field on bone metabolism [24] is extremely helpful for osteosarcoma patients with pathological fracture which leads to poor prognosis [25, 26].

Considering osteosarcoma is especially prevalent in children and young adults during quick osteoblastic differentiation [1, 2], unstable RB gene and p53 gene are commonly involved in this malignant transformation process [27]; we hypothesize that nsPEF affects osteosarcoma growth by targeting the Wnt/β-catenin signaling pathway, a key signaling cascade involved in osteosarcoma pathogenesis. Here, we investigate nsPEF-induced changes on human osteosarcoma MG-63 cells to determine (1) the dose-effect relationship and time-effect relationship of nsPEF on osteosarcoma cell growth and apoptosis induction and (2) the nsPEF effect on the osteosarcoma cell; osteoblast specific gene and protein expression (receptor activator of NF-κB ligand (RANKL) and osteoprotegerin (OPG)) were measured along with the production of the pro-inflammatory cytokine tumor necrosis factor α (TNF-α).

## Materials and methods

### Cell lines and cell culture

MG-63 human osteosarcoma cells were purchased from the Cell Bank of Chinese Academy of Sciences (Shanghai, China), cultured in Dulbecco’s Modified Eagle’s medium (DMEM, Gibco Invitrogen, Carlsbad, CA, USA) supplemented with 10 % fetal bovine serum (FBS, SAFC Biosciences, Lenexa, KS, USA), 100 units/mL penicillin, and 100 mg/mL streptomycin (Sigma, Aldrich, St. Louis, MO, USA). Cells were kept in a humidified atmosphere of 5 % CO2 at 37 °C.

### The nsPEF treatment and dose-effect exam

The nsPEF treatment system was made by Leibniz Institute for Plasma Science and Technology, Germany, and an nsPEF generator with duration of 100 ns was applied. Varied electric fields were released in a cell treatment system from 10 to 60 kV/cm. Waveforms were monitored with a digital phosphor oscilloscope (DPO4054, Tektronix, USA) equipped with a high voltage probe (P6015A, Tektronix, USA). MG-63 human osteosarcoma cells were harvested with trypsin and resuspended in fresh DMEM with 10 % FBS to a concentration of 5.0 × 10^6^ cells/mL. Five hundred microliters of cell suspension were placed into a sterile electroporation cuvette (Bio-Rad, US, 0.1-cm gap). Cells were exposed to 100 pulses at 0, 10, 20, 30, 40, 50, and 60 kV/cm electric field strengths, respectively. Under the 50 kV/cm electric field strength, the different pulse numbers were applied (0, 6, 12, 18, 24, and 30 pulses). The experiments were repeated for three times. After incubation for 24 h, cells were calculated by Cell Counting Kit-8 (CCK-8) assay (Dojindo Laboratories, Kumamoto, Japan).

### Measurement of apoptosis with TUNEL assay, Hoechst stain, and flow cytometry

At different hours after nsPEF treatment (40 kV/cm, 30 pulses), the treated cells were incubated for 0, 3, 12, 24, and 48 h to determine single-cell apoptosis using the assay of terminal deoxynucleotidyl transferase (TdT)-mediated dUTP nick-end labeling (TUNEL) with In Situ Cell Death Detection Kit (Millipore, USA) and Hoechst stain kit (Beyotime, Shanghai, China) according to the manufacturer’s instruction, as previously described [14]. Under different electric field strengths and with different pulses, the treated cells were incubated for 24 h to detect cell apoptosis by Annexin V-FITC Apoptosis Detection Kit (BD Biosciences). The cell cycle was also analyzed as previously described [14].

### Reverse-transcription polymerase chain reaction

Reverse-transcription polymerase chain reaction (RT-PCR) was performed for assessing the expression of OPG, RANKL, and TNF-α. Glyceraldehyde-3-phosphate dehydrogenase (GAPDH), a house keeping gene, was used as the internal control to calculate the comparative expression. Total RNA was extracted using TRIzol reagent (Sangon, Shanghai, China). The first strand cDNA synthesis from 1 mg of RNA was performed using SuperScript II Reverse Transcriptase (Invitrogen) and Oligo dT primer (Promega, Madison, WI, USA) according to the manufacturer’s instructions. PCR was performed using the oligunucleotides listed as the following. The specific primers were made by Sangon, Shanghai, China, which were listed as the following: RANK: F: CAGGAGACCTAGCTACAGA, R: CAAGGTCAAGAGCATGGA, 95 °C, 1 min; 55 °C, 1 min; 72 °C, 1 min; OPG (264 bp): F: AGTGGGAGCAGAAGACAT, R: TGGA CCTGGTTACCTATC, 95 °C, 1 min; 57 °C, 1 min; 72 °C, 1 min; TNF-α: F: GTGGCAGTCTCAAACTGA, R: TATGGAAAGGGGCACTGA, 94 °C, 40 s; 55 °C, 40 s; 72 °C, 40 s; GAPDH: F: CAG CGACACCCACTCCTC, R: TGAGGTCCA CCACCCTGT, 94 °C, 1 min; 57 °C, 1 min; 72 °C, 1 min.

### Western blotting analysis

MG-63 cells (5 × 10^5^) were plated and treated with different doses of nsPEF. Cells were then lysed with a lysis buffer and then quantified. The equal amounts of protein were loaded, and electrophoresis was applied on a 12 % sodium dodecyl sulfate-polyacrylamide gel electrophoresis mini-gel. Proteins were transferred to a PVDF membrane and blocked with casein PBS and 0.05 % Tween-20 for 1 h at room temperature. Membranes were incubated with mouse monoclonal OPG, anti-OPG (1:500), RANKL (1:200), TNF-α (1:300), GAPDH (1:1000) antibodies which were purchased from Santa Cruz (Santa Cruz Biotechnology, Santa Cruz, CA, USA). Horseradish peroxidase-conjugated secondary antibody was purchased from Zhongshan (Zhongshan Golden Bridge, Beijing, China.). The protein expression was visualized with enhanced chemiluminescence reagent (ECL kit, Amersham, UK).

### Statistical analysis

Statistical significance was determined using Student’s *t* test, using SPSS 13.0. *P* < 0.05 was considered to indicate a statistically significant result.

## Results

### NsPEF parameter optimizing by CCK-8 and flow cytometry

**Fig. 1:**
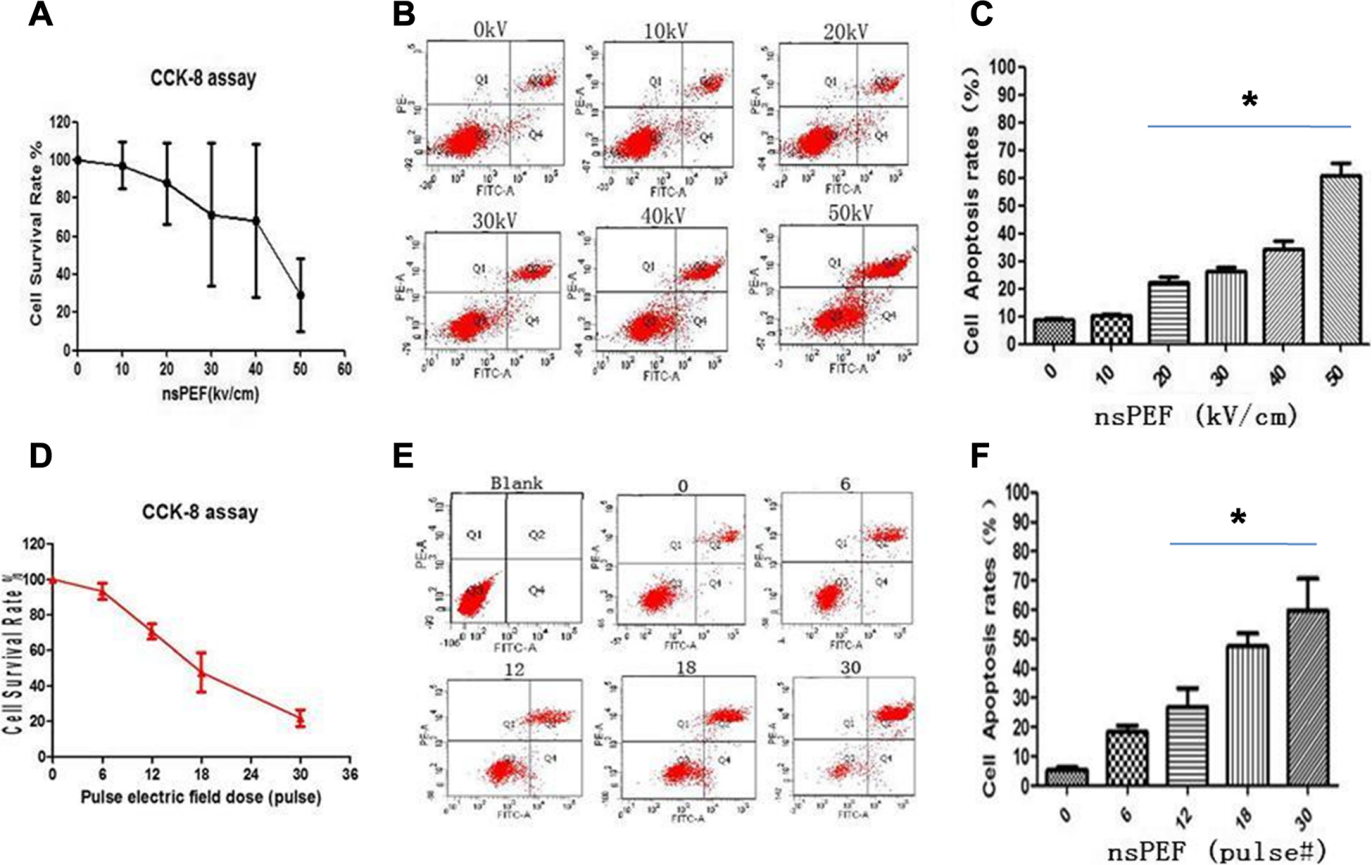
NsPEF treatment parameter optimizing by CCK-8 and flow cytometry. After 24 h post nsPEF, CCK-8 assay was used to calculate the IC50 values under different electric field strengths (**a**) and different pulse numbers (**d**). The flow cytometry was used to detect apoptotic histograph under different electric field strengths (**b**) and different pulse numbers (**e**) as well as the apoptotic cell rate under different electric field strengths (**c**) and different pulse numbers (**f**). There was significant (*P* > 0.001) growth inhabitation when electric field strength was 30, 40, and 50 kV/cm (**a**) and when pulse number was 30 (1D) vs control. The apoptotic cell rate is significantly increased when electric field strength was 40–50 kV/cm (**b**, **c**) and when pulse number was 30 (**e**, **f**)

CCK-8 assay was used to calculate the IC50 values, and flow cytometry was used to detect apoptosis. There were significant growth inhibition and apoptosis induction in a dose-dependent manner following nsPEF treatment for 24 h. MG-63 cell growth was inhibited in an electric field strength- and pulse number-dependent manner. There was significant (*P* > 0.001) growth inhibition when electric field strength was 40–50 kV/cm (Fig. 1a) and when pulse number was 30 (Fig. 1d) vs control. Cells were treated by nsPEF and then incubated for 24 h. Apoptotic and dead cells were analyzed by flow cytometry using dual staining with propidium iodide (PI) and Annexin V-FITC. NsPEF induced viable apoptotic cells stained with Annexin. The apoptotic cell rate is significantly increased when electric field strength was 40–50 kV/cm (Fig. 1b, c) and when pulse number was 30 (Fig. 1e, f).

### Apoptosis induction at different times post nsPEF treatment

**Fig. 2:**
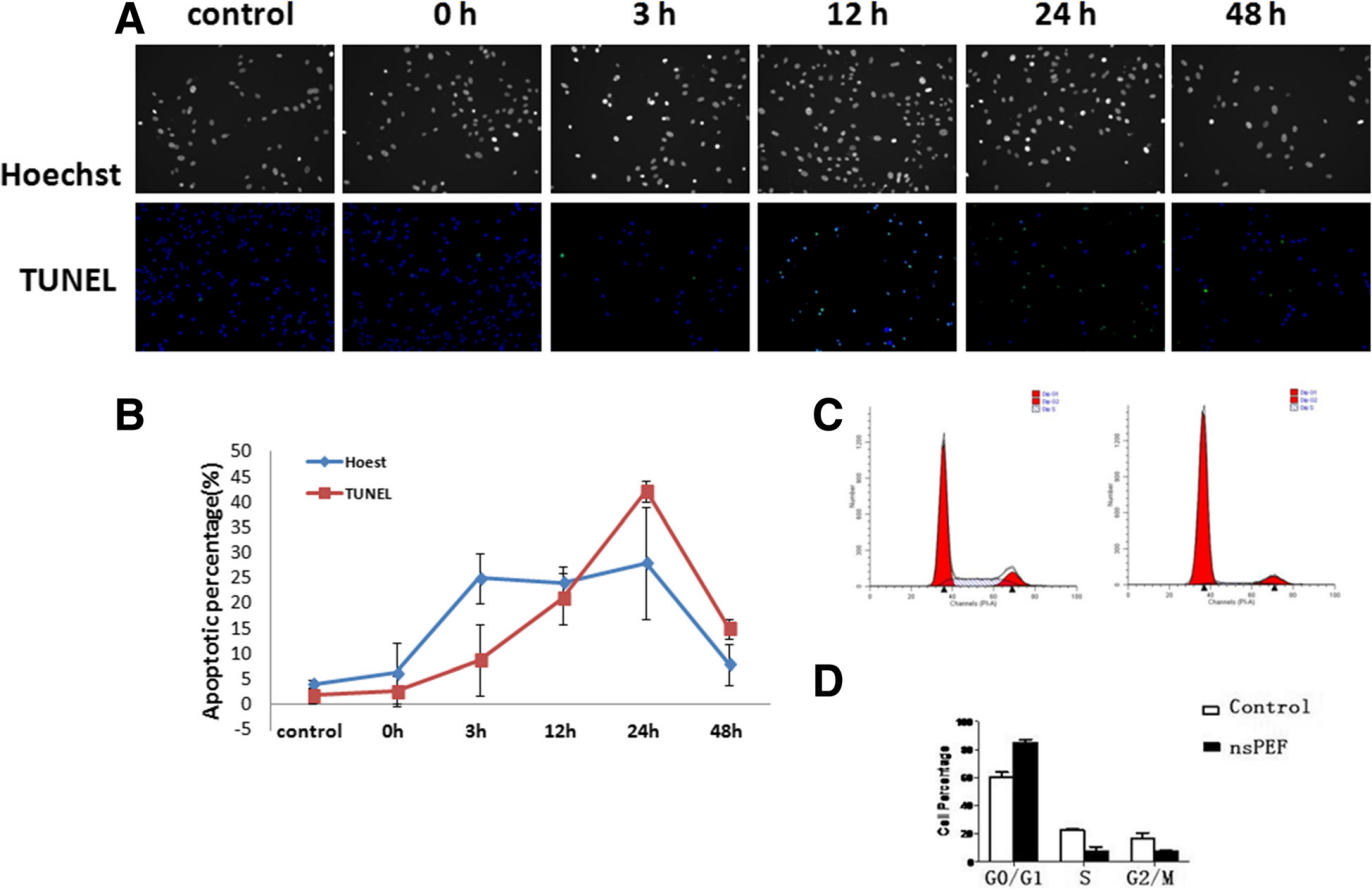
Apoptosis induction at different times post nsPEF treatment. After 40 kV/cm and 30 pulses of nsPEF treatment, the control and treated cells were stained with Hoechst 33528 (**a** *upper lane*) and TUNEL (**a** *lower lane*). The statistical analysis of the positive apoptotic cells were counted and shown in (**b**) at different hours (0, 3, 12, 24, and 48 h). The apoptotic cells were significant in 24 h post nsPEF treatment. The cell cycle was analyzed by flow cytometry (**c**) and statistically analyzed in (**d**), which indicates that nsPEF arrest cells in the G0/G1 phase

To determine the effects of nsPEF on the induction of apoptosis in MG-63 cells, the Annexin V assay was performed. After 40 kV/cm and 30 pulses of nsPEF treatment, the control and treated cells were stained with Hoechst 33528 (Fig. 2a upper lane) and TUNEL (Fig. 2a lower lane). The statistical analysis of the positive apoptotic cells were counted and shown in Fig. 2b at different hours (0, 3, 12, 24, and 48 h). Apoptotic cells induced by nsPEF treatment were recognized by terminal deoxynucleotidyl transferase (TdT)-mediated dUTP nick-end labeling (TUNEL), detecting DNA fragmentation by labeling the terminal end of nucleic acids. The number or percentages of apoptotic cells detected following nsPEF treatment was shown in Fig. 2b. The quantitative analysis showed the percentages of apoptotic cells detected following nsPEF treatment which were 2.6 % (0 h), 8.8 % (3 h), 21 % (12 h), 42 % (24 h), and 15 % (48 h) without nsPEF treatment. The apoptotic induction 12 and 24 h post nsPEF treatment showed significance (*P* = 0.01243, 0.00081, respectively, vs control). The cell cycle was analyzed by flow cytometry (Fig. 2c) and statistically analyzed in Fig. 2d, which indicates that nsPEF arrest cells in the G0/G1 phase (Fig. 2d).

### The effect of nsPEF on OPG/RANKL, TNF-α gene, and protein expression

**Fig. 3:**
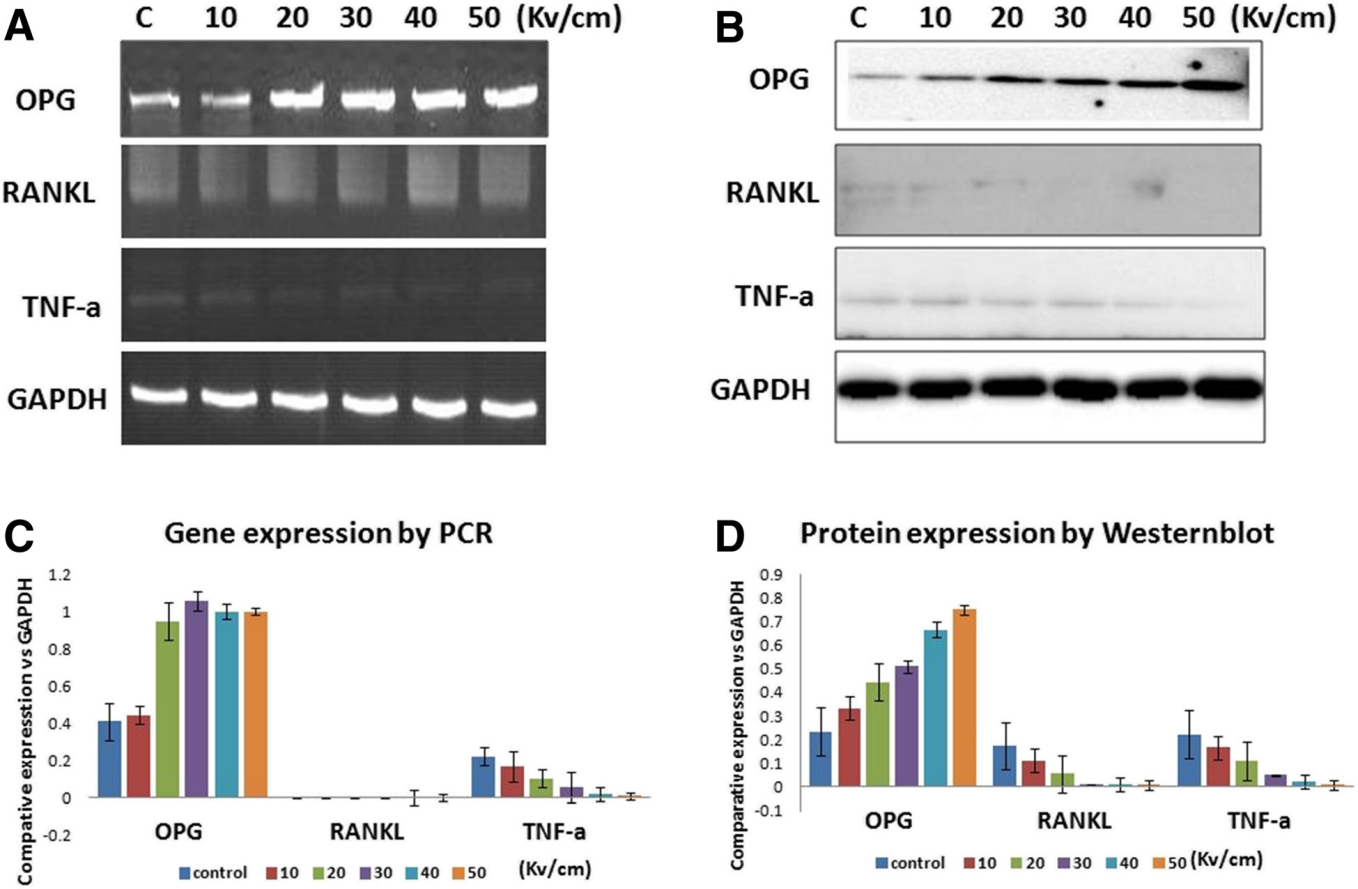
The nsPEF effect on gene and protein expression. With 30 pulses, 24 h post treatment, PCR and western blot were used to determine the different electric field strengths on cell OPG/RANKL, TNF-α gene (**a**), and protein expression (**b**). NsPEF significantly increased OPG transcription and protein expression at 20–50 kV/cm (**a**, **c**). RANKL was almost undetectable both in the control and nsPEF-treated MG-63 cells (**a**, **d**). NsPEF slightly down-regulated TNF-α (**a**, **d**)

With 30 pulses, 24 h post treatment, PCR and western blot were used to determine the different electric field strengths on cell OPG/RANKL, TNF-α gene (Fig. 3a), and the corresponding protein expression (Fig. 3b). NsPEF significantly increased OPG transcription and protein expression at 20–50 kV/cm (Fig. 3a, c). RANKL was almost undetectable both in the control and nsPEF-treated MG-63 cells (Fig. 3a, c). NsPEF slightly down-regulated TNF-α (Fig. 3a, c). The OPG is important in the regulation of bone formation. PCR results showed that the nsPEF-treated cells demonstrated a significantly up-regulation of OPG transcription. Western blot analysis confirmed that nsPEF stimulated osteoprotegerin protein production in the MG-63 cells.

## Discussion

The primary bone malignancy osteosarcoma is still a challenge for orthopedics. For patients who are not suitable for radical resection, the minimal invasive ablation techniques can be used as an alternative to surgery. NsPEF has been proved to be a novel non-thermal ablation method which can activate a protection immune response [21–23]. According to the Clinical Practice Guidelines in Oncology of the National Comprehensive Cancer Network (NCCN), local ablation can be used for curative or palliative intent, either alone or in combination with immunotherapy or chemotherapy [11]. The effect of systemic chemotherapy may be enhanced by the physiological changes produced by ablation [11]. Furthermore, ablation can sometimes be used as a complement to surgery [13].

A number of studies have demonstrated that local ablation is effective in osteosarcoma [28–30]. To our best knowledge, the application of nsPEF in osteosarcoma has never been reported. The bone-related tumor study is extremely important because many solid tumors tend to have metastasis in bones. The present study applies a new ablation methodology in osteosarcoma and identifies its molecular target. Our data suggest that nsPEF had direct effects on osteosarcoma cells, including the inhibition of tumor cell proliferation and induction of apoptosis. These results are consistent with previous reports. NsPEF inhibits cell proliferation and induces apoptosis in tumor cells [11, 16].

The development of osteoclasts is controlled by cytokine synthesized by osteoblasts like receptor activator of NF-κB ligand (RANKL), osteoprotegerin (OPG), and tumor necrosis factor α (TNF-α) [31].The extension of the current study is the investigation of nsPEF’s effect on bone resorption when nsPEF is in its ablation dosage. OPG is a member of the tumor necrosis factor receptor family. It has multiple biological functions such as regulation of bone turnover. OPG can block the interaction between RANKL and the RANK receptor [31]. NsPEF increased OPG expression in MG-63 in in vitro assays. Our data indicate that nsPEF up-regulated the OPG expression. Bone remodeling can be assessed by the relative ratio of OPG to RANKL [32]. NsPEF had no effect on RANKL expression. Defined as a potent bone-resorbing factor, TNF-α is responsible for stimulating bone resorption. TNF-α exerts its osteoclastogenic effect by activating NF-κB with RANKL [33]. Our results show that in osteosarcoma MG-63, in addition to apoptosis induction, nsPEF can regulate bone metabolism through adjusting OPG/RANKL ratio.

TNF-α expression still needs further investigation due to the weak expression. But, it is the key cytokine that we assume which would change the local inflammatory microenvironment in the ablation zone.

### The limit of the current study

In this in vitro study, the MG-63 osteosarcoma cell line is used as a model system. Therefore, results obtained from cultured cells only gave hints for the nsPEF treatment of osteosarcoma. The current results need to be tested in an in vivo osteosarcoma model, e.g., MG-63 cell xenografts.

## Conclusion

NsPEF can be considered as a potential therapeutic intervention to suppress bone remodeling and osteoclast activity involved in osteosarcoma. Further in vivo studies are required to optimize the dosing regimen of nsPEF to fully study its antitumor potential in the bone microenvironment.
